# Bibliography

**Published:** 1905-05-15

**Authors:** 


### BIBLIOGRAPHY.

A MANUAL OF MECHANICAL DENTISTRY.—The second edition of this book by Dr. George W. Warren, of the Pennsylvania Dental College, is just received. This small work attempts to cover the whole field of what its author is pleased to term Mechanical Dentistry. His evident definition is the entire subject of prosthetic dentistry including, plate, crown and bridge-work, orthodontia, metallurgy, obturators and dental splints. This is a fairly big subject to set forth in a manual of 250 pages of the 12mo. size, and using large sized type. The book is also very well illustrated with a large number of good drawings. The book is intended for the use of students and necessarily contains a mere statement of the most accepted methods of handling these subjects. The author is to be commended for his ability to get so many of the essential principles in so small a work. We question the propriety of such condensation even for student's use. The object, of course, is to save the student the expense of the more costly works. For a real student such a work gives no satisfactory statement of any subject. If these works are to be used as text-books they should have abundant reference notes to the more ample literature on each subject, so that a student desiring to put intellectual effort into his work, would have such direction as would enable him to study to his satisfaction any subject in which he might become interested. We should split the work up into at least three books, and drop the name "Mechanical Dentistry," which is indefinte and inappropriate. It is published by the well-known P. Blakiston's Son & Co.

---

ORAL PATHOLOGY AND THERAPEUTICS.—This is a new book by Dr. Elgin MaWhinney, Professor of Dental Pathology and Therapeutics in the Northwestern University Dental School, and is published by the Consolidated Dental Manufacturing Co., of New York City. It is an octavo size of 233 pages. The author, in his preface, states that it is his desire to place this subject on a more logical or scientific basis than exists at the present time. He takes up the subject of dental caries and follows the usual sequences to alveolar abscesses. necrosis, etc., each subject being considered in a chapter by itself. The author gives his own interpretation of usual and typical conditions, and outlines his therapeutic treatment. The style of description is informal rather than pedagogic. As would be expected, in so small a work, no comprehensive pathological discussion is possible, and the treatment, while generally scientific, will not meet the approval of every one. The book bears evidence of too much haste in the preparation of its matter as well as in proof reading, and yet it is most interesting reading. There are many useful and important methods or details of practice that are not even mentioned which should be incorporated in a text-book on this subject. The chapter on obtundents, for instance, leaves out cataphoresis, injection by the hypodermic syringe, and other valuable methods entirely. The book needs a better index there is no drug index of any kind.

